# Neoadjuvant Therapy and the Evolving Management of Resectable Advanced Melanoma

**DOI:** 10.3390/cancers18121978

**Published:** 2026-06-18

**Authors:** Nikolaos Papadopoulos, Michele Del Vecchio, Andrea Spagnoletti, Jacopo Pigozzo, Luisa Piccin, Alessandro Minisini, Federico Pravisano, Gabriele Roccuzzo, Paolo Fava, Carolina Cimminiello

**Affiliations:** 1 Division of Early Drug Development for Innovative Therapies, European Institute of Oncology, Scientific Institute for Research, Hospitalization and Healthcare (IRCCS), 20141 Milan, Italy; nikolaos.papadopoulos@ieo.it; 2 Department of Oncology and Hemato-Oncology (DIPO), University of Milan, 20122 Milan, Italy; 3 Unit of Melanoma Medical Oncology, Department of Medical Oncology, Fondazione IRCCS Istituto Nazionale dei Tumori, 20133 Milan, Italy; michele.delvecchio@istitutotumori.mi.it (M.D.V.); andrea.spagnoletti@istitutotumori.mi.it (A.S.); 4 Medical Oncology 2, Veneto Institute of Oncology IOV-IRCCS, 35128 Padua, Italy; jacopo.pigozzo@iov.veneto.it (J.P.); luisa.piccin@iov.veneto.it (L.P.); 5 Department of Medical Oncology, Santa Maria della Misericordia Academic Hospital, Azienda Sanitaria Universitaria Friuli Centrale (ASUFC), 33100 Udine, Italy; alessandro.minisini@asufc.sanita.fvg.it (A.M.); federico.pravisano@asufc.sanita.fvg.it (F.P.); 6 Department of Medical Sciences, Section of Dermatology, University of Turin, 10124 Turin, Italy; gabriele.roccuzzo@unito.it (G.R.); paolo.fava@unito.it (P.F.); 7 Division of Clinical and Experimental Oncology and Melanoma Immunotherapy, European Institute of Oncology, Scientific Institute for Research, Hospitalization and Healthcare (IRCCS), 20141 Milan, Italy

**Keywords:** neoadjuvant therapy, PRADO, NADINA, biomarkers

## Abstract

Melanoma has long represented a unique challenge in oncology. In its early stages, it can often be cured with surgery alone, resulting in excellent survival outcomes. Its biology is complex and heterogeneous, often including mutations such as BRAF and NRAS, which partly explain its high immunogenicity but also its capacity to relapse. For many decades, the standard approach in resectable stage III melanoma was surgery first, followed by adjuvant therapy. In the last decade, the development of immune checkpoint inhibitors created the opportunity to treat patients before surgery. The neoadjuvant approach is based on the hypothesis that treatment of the intact tumor may enhance antitumor immune priming and activation. The first prospective neoadjuvant studies, including OpACIN and OpACIN-neo, showed high pathological response rates with ipilimumab plus nivolumab and introduced pathological response as an early marker of long-term benefit. The PRADO study showed that treatment response could guide the extent of surgery required. The SWOG S1801 trial demonstrated better event-free survival with perioperative pembrolizumab compared to adjuvant therapy alone. Lastly, the phase III NADINA trial showed that neoadjuvant ipilimumab plus nivolumab followed by response-adapted adjuvant therapy significantly improves outcomes. Biomarkers such as PD-L1, IFN-γ signature, tumor mutational burden and imaging (PET scan) appear promising to predict response, but none have been sufficiently validated for routine clinical practice.

## Simple Summary

Melanoma is a skin cancer that, in early stages, can often be cured with surgery alone, but even complete removal of the primary tumor does not eliminate the risk of disease recurrence. In recent years, immunotherapy before surgery has become an increasingly important treatment strategy, with the idea that treating the tumor while it remains intact may induce a stronger immune response and reduce the risk of relapse. Several clinical trials have shown encouraging results, suggesting in some cases even less extensive surgery. At the same time, researchers are seeking to identify biological markers from tumor samples or imaging that could help determine which patients are most likely to benefit from this strategy. The aim of this review is to summarize the current evidence and highlight how earlier treatment could improve clinical outcomes while moving towards a more personalized approach.

## 1. Introduction

**Figure 1:**
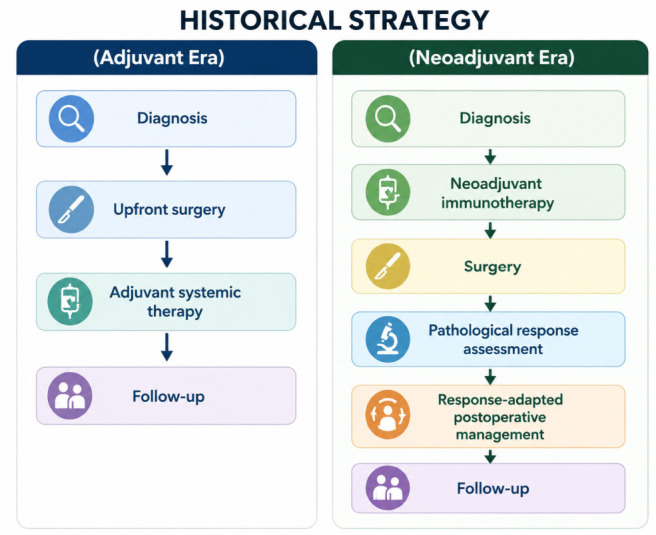
Historical versus modern treatment paradigm in resectable stage III melanoma.

Melanoma is responsible for approximately 60,000 deaths annually worldwide. Its incidence has been increasing in recent decades, especially in populations with lighter phototypes. Biologically, melanoma is characterized by a high molecular heterogeneity, frequently showing alterations in signaling pathways such as MAPK and alterations in BRAF or NRAS genes, which partly explain its aggressiveness. Prognosis in melanoma is strongly related to initial stage of diagnosis. Patients with localized disease tend to have an excellent outcome after surgery, with survival rates even approaching 99% in early-stage melanoma, while outcomes progressively worsen in more advanced stages. Importantly, even when the disease appears completely removable, the risk of recurrence may remain substantial, suggesting that microscopic dissemination may occur earlier than clinically evident. Resectable melanoma refers to the stage in which all visible tumor lesions can still be removed surgically with curative intent. This definition mainly includes patients with stage III melanoma, with regional lymph node involvement, in-transit metastases, and a small group of selected patients with limited stage IV disease in whom metastases are technically operable. Traditionally, such patients were treated with upfront surgery followed by adjuvant systemic therapy [1]. Adjuvant anti-PD-1 immunotherapy has demonstrated improvement in recurrence-free survival (RFS) and distant metastasis-free survival (DMFS), becoming an important component of modern melanoma management. However, a substantial proportion of patients will still experience disease recurrence, highlighting that melanoma can also behave as a systemic disease in these intermediate stages (Figure 1) [2]. Over the past decade, the introduction of immune checkpoint inhibitors and targeted therapies has profoundly modified the therapeutic landscape of melanoma. A new concept emerged, shifting interest toward using systemic treatment earlier in the course of the disease. Trials such as PRADO and NADINA have started to question the traditional sequence of surgery followed by systemic therapy in patients with resectable advanced melanoma. Several recent reviews have summarized the biological rationale and emerging biomarkers associated with neoadjuvant immunotherapy in melanoma. The aim of this review is not only to summarize current evidence but also to discuss the evolution from the adjuvant era to modern neoadjuvant strategies, the clinical implications of NADINA, and the emerging role of biomarkers in treatment personalization and routine clinical application.

## 2. Materials and Methods

This narrative review was conducted through a literature search of PubMed/MEDLINE. Relevant studies published in English were identified using combinations of the following keywords: “melanoma”, “neoadjuvant therapy”, “immunotherapy”, “checkpoint inhibitors”, “ipilimumab”, “nivolumab”, “pembrolizumab”, “SWOG S1801”, “NADINA”, “PRADO”, “OpACIN”, “pathological response”, “biomarkers”, and “circulating tumor DNA”.

Priority was given to prospective clinical trials, randomized studies, landmark publications, and recent reviews focusing on resectable stage III and selected stage IV melanoma. Additional references were identified through manual review of bibliographies from relevant articles. The selection of studies was based on their scientific relevance, clinical impact, and contribution to the evolving understanding of neoadjuvant treatment strategies and biomarker development in melanoma.

## 3. Neoadjuvant Approach in Resectable Melanoma: From Early Evidence to the SWOG and NADINA Experience

The move from adjuvant to neoadjuvant therapy in resectable melanoma did not happen overnight. Before the emergence of neoadjuvant strategies, the management of resectable stage III melanoma was based on upfront surgery followed by adjuvant systemic therapy. The modern adjuvant era was established by pivotal trials such as COMBI-AD, KEYNOTE-054, and CheckMate 238, which demonstrated significant reductions in recurrence risk with BRAF/MEK-targeted therapy and anti-PD-1 immunotherapy. These studies established adjuvant therapy as the standard of care for resected stage III melanoma and substantially improved recurrence-free survival outcomes. Nevertheless, a considerable proportion of patients continued to relapse despite optimal postoperative treatment, providing the rationale for exploring whether systemic therapy could be delivered more effectively before surgery.

The clinical frustration with the limits of the post-operative strategy and the results of adjuvant immunotherapy provided the rationale for exploring preoperative treatment strategies. Although adjuvant systemic therapies have significantly improved RFS and DMFS, mature overall survival data have been less consistent across studies, and the optimal sequencing of systemic therapy remains an area of active investigation. In this context, the concept of preoperative systemic therapy became increasingly attractive. The biologic rationale was compelling. Immune checkpoint inhibitors rely on the presence of tumor antigens and on the pre-existing tumor-reactive T cells. When surgery is performed as the initial treatment, the tumor bulk is essentially eliminated, removing with it a substantial portion of the tumor-associated immune microenvironment. Performing immunotherapy while the tumor is still in place can generate a broader T-cell priming, activation, and clonal expansion [2]. Translation of this hypothesis into clinical practice, already supported by preclinical models, made melanoma one of the first solid tumors in which this concept was tested in a structured way. Early investigations included intralesional approaches such as talimogene laherparepvec (T-VEC) [3]. The long-term follow-up of neoadjuvant T-VEC plus surgery demonstrated improvements in RF rate and OS compared to surgery alone. However, the magnitude of benefit remained limited in comparison to modern checkpoint inhibitor-based regimens. The first clinical evidence emerged from early-phase studies with neoadjuvant checkpoint blockade, particularly the OpACIN and OpACIN-neo trial, which explored the use of ipilimumab and nivolumab in patients with macroscopic stage III melanoma. These studies not only showed that neoadjuvant immunotherapy was feasible but also introduced a concept that later became a central objective of subsequent trials, that pathologic response may act as an early surrogate of long-term outcome. In these early experiences, pathologic response rates were remarkably high, around 74% to 78%, and patients who achieved a pathologic response had excellent RFS at 2 years, in the range of 94% to 100%. The OpACIN-neo trial also defined the regimen that would later be adopted in subsequent studies: ipilimumab 1 mg/kg and nivolumab 3 mg/kg for two cycles. This dosing schedule provided a favorable balance between efficacy and toxicity. Grade 3–4 toxicities with this schedule were generally reported in roughly 27% to 30% of patients [4,5].

A major step forward came with the PRADO trial, a single-arm phase II study that showed how neoadjuvant treatment could personalize the overall management of stage III melanoma. In PRADO, 99 patients with clinical stage III nodal melanoma received 2 cycles of neoadjuvant ipilimumab plus nivolumab. At week 6, treatment management was adapted according to pathological response assessed in the index lymph node. The overall pathologic response rate was around 72%, including a major pathologic response (MPR), defined as ≤10% viable tumor, in 61% of patients. Grade 3–4 toxicity in the first 12 weeks occurred in 22% of patients. The key finding of this study was that therapeutic lymph node dissection was omitted in 59 of 60 patients with major pathologic response, leading to lower surgical morbidity. At 24 months, RFS was 93%, and DMFS was 98% in the MPR group, compared to 71% and 76% in patients with pNR. PRADO was not practice-changing as it was not a phase 3 randomized study, but it created a new clinical framework in which early tumor response could guide the need for post-operative treatment. More recently, the 5-year update of PRADO reinforced this message [6,7]. The estimated 5-year EFS was 71%, RFS was 74%, DMFS was 79%, and OS was 86%. The outcomes were especially favorable in patients with major pathologic response, with 5-year RFS of 86% and 5-year DMFS of 91%, whereas results were much worse in patients with pPR or pNR. These long-term data supported the concept that pathologic response could function not only as a short-term marker, but also as an indicator of durable benefit [6,7]. Pathological complete response (pCR) refers to the absence of viable tumor cells in the surgical specimen. Major pathological response (MPR) is generally defined as ≤10% residual viable tumor, while partial pathological response (pPR) corresponds to >10% and ≤50% viable tumor. Patients with >50% viable tumor are classified as having pathological non-response (pNR). Because different studies may emphasize different pathological endpoints, careful interpretation is required when comparing results across trials. One of the most important concepts emerging from PRADO was that pathological response could potentially guide the extent of surgery. Patients achieving major pathological response were able to avoid completion therapeutic lymph node dissection without compromising oncologic outcomes. Although this strategy requires further validation before widespread adoption, it introduced the possibility that surgery may eventually become more personalized and less extensive. Although the results of PRADO were highly encouraging, several limitations should be acknowledged. The study was a single-arm phase II trial without a randomized comparator, thus limiting conclusions regarding survival benefit. Also, the treatment decisions were guided by pathological response, introducing potential selection effects when comparing outcomes between the responders and the non-responders. In addition, the relatively limited sample size of the study and the enrollment of patients in specialized melanoma centers may also affect the generalizability of the findings. Nevertheless, PRADO provided important proof-of-concept evidence supporting response-adapted management and established pathological response as a clinically impactful endpoint [6,7].

The first randomized study to demonstrate that neoadjuvant immunotherapy could outperform adjuvant therapy was SWOG S1801, a phase II trial that enrolled patients with clinically detectable, stage IIIB to IVC melanoma that was considered resectable. The patients were randomized to three cycles of pembrolizumab before surgery, followed by 15 adjuvant cycles, or to up-front surgery, followed by 18 adjuvant cycles of pembrolizumab.

SWOG S1801 represented the first randomized evidence supporting the clinical benefit of a neoadjuvant approach. At a median follow-up of 14.7 months, EFS was significantly longer in the neoadjuvant–adjuvant group. In the landmark analysis, 2-year EFS was 72% with the neoadjuvant approach versus 49% with adjuvant therapy. Notably, the low percentage of treatment-related toxicity with adverse events of grade 3 or higher occurring in 12% of patients in the neoadjuvant–adjuvant group and 14% in the adjuvant-only group. SWOG S1801 provided randomized evidence that timing matters in melanoma immunotherapy, but at the same time, it still left several important questions unanswered. The regimen was pembrolizumab alone, which was attractive in terms of safety, but may not represent the most active neoadjuvant regimen currently available. In addition, the trial included both stage III and selected stage IV patients, a choice that reflects real-world clinical complexity but also complicates interpretation of the results. The biological behavior, recurrence patterns, and prognosis of these groups may differ substantially, making it difficult to determine whether the magnitude of benefit was uniform across all disease stages [8].

These limitations ultimately provided the rationale for the phase III NADINA trial. NADINA enrolled 423 patients at least 16 years of age with resectable, macroscopic stage III melanoma. These patients had pathologically proven nodal metastases that were palpable, PET-positive, or measurable on imaging according to RECIST. Patients could also have up to three in-transit metastases. They were randomized to two arms: either receiving two cycles of neoadjuvant ipilimumab 80 mg plus nivolumab 240 mg every 3 weeks followed by surgery, or to upfront surgery followed by 12 cycles of adjuvant nivolumab. The most innovative aspect of NADINA was that the neoadjuvant arm was response-adapted. Patients with an MPR, defined as ≤10% residual viable tumor, did not receive adjuvant treatment. Those with pPR or pNR received adjuvant therapy tailored by BRAF status: dabrafenib plus trametinib for BRAF V600E/K mutant disease, or nivolumab for BRAF wt patients.

The efficacy results were highly encouraging. The NADINA trial recorded an estimated 12-month EFS of 83.7% in the neoadjuvant group versus 57.2% in the adjuvant group. The hazard ratio for progression and recurrence was 0.32 (99.9% CI, 0.15 to 0.66; *p* < 0.001), corresponding to a 68% reduction in the risk of progression or recurrence. In the neoadjuvant arm, 59.0% of patients achieved a MPR, 8% had a pPR, 26.4% had pNR, and 2.4% had progression before surgery; in 4.2%, surgery had not yet been performed or was omitted at the time of the analysis. These numbers are very consistent with the earlier PRADO experience. In essence, NADINA confirmed in a phase III setting what PRADO had strongly suggested: pathologic response is tightly linked to outcome. In the neoadjuvant group, the estimated 12-month RFS was 95.1% in patients with MPR, 76.1% in those with pPR, and only 57% in those with pNR.

The results of NADINA suggest that a neoadjuvant strategy may offer several potential advantages. Firstly, it improves EFS compared with the standard adjuvant approach. Secondly, it identified that a large subgroup of patients, approximately 60% of patients who achieve an MPR, were therefore able to avoid further adjuvant systemic therapy. Thirdly, it can identify non-responders early during treatment, allowing earlier therapeutic planning.

**Figure 2:**
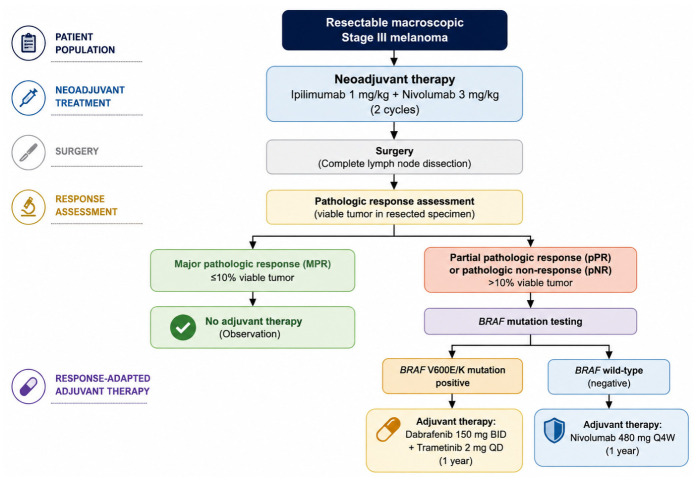
Response-adapted treatment strategy in the Nadina Trial.

Regarding toxicity, in NADINA, 29.7% of patients in the neoadjuvant group experienced a grade 3 or higher adverse event versus the 14.7% in the adjuvant group. Endocrinopathies related to systemic treatment were also more common with the neoadjuvant strategy, occurring in 30.7% versus 9.9%. However, surgery-related grade 3 or higher adverse events showed a clear similarity between groups: 14.1% in the neoadjuvant arm and 14.4% in the adjuvant arm. Notably, no treatment-related deaths were reported in the neoadjuvant group, whereas one patient in the adjuvant group died from immune-related pneumonitis (Figure 2) [9,10,11].

While the results of SWOG S1801 and NADINA have significantly advanced the field, several aspects should be considered when interpreting their findings. To start, the study populations differed substantially. SWOG S1801 enrolled patients with stage IIIB to IV resectable melanoma, whereas NADINA focused exclusively on macroscopic stage III disease. These differences limit direct comparisons between the studies and may partially explain variations in outcomes. Furthermore, the studies used different treatment strategies, patient populations, and follow-up durations. While both trials support the application of neoadjuvant treatment, differences in design preclude direct comparisons regarding the magnitude of benefit.

Second, follow-up remains relatively limited, particularly for NADINA. Although the EFS benefit observed in both studies is compelling, longer follow-up will be necessary to determine the durability of these responses and their eventual impact on overall survival. This is especially relevant in melanoma, where late recurrences can occur and where overall survival remains the most clinically meaningful endpoint.

Another important consideration is external validity. Both trials were conducted in experienced centers with substantial expertise in melanoma management, multidisciplinary decision-making, and treatment-related toxicity management. Whether similar outcomes can be consistently reproduced in broader real-world populations remains an open question because implementation of neoadjuvant strategies currently varies across institutions and healthcare systems, reflecting differences in multidisciplinary expertise, access to specialized melanoma care, and the evolving nature of international guidelines.

Finally, treatment-related toxicity should be considered when translating these findings into clinical practice. The impressive efficacy observed with neoadjuvant ipilimumab plus nivolumab in NADINA came at the cost of higher rates of grade ≥ 3 immune-related adverse events. Consequently, patient selection remains crucial. Elderly patients, individuals with significant comorbidities, pre-existing autoimmune conditions, or reduced performance status may not derive the same risk-benefit ratio observed in clinical trial populations. Future studies should focus not only on improving efficacy but also on identifying patients most likely to benefit from treatment intensification.

In conclusion, the collective experience from OpACIN, PRADO, SWOG S1801, and NADINA has generated several important concepts that extend beyond the individual results of each study. Firstly, they demonstrated that pathological response is one of the strongest prognostic markers identified in resectable melanoma. Secondly, they established that treatment timing itself may influence clinical outcomes, supporting the rationale for using immune checkpoint blockade before surgery. Thirdly, they introduced the concept of response-adapted management, in which postoperative treatment decisions may be guided by pathological findings rather than by baseline stage alone (Table 1).

## 4. Role of Biomarkers in Clinical Trials of Resectable Melanoma

As neoadjuvant therapy has emerged as an increasingly important component of melanoma management, biomarkers are evolving from a predominantly translational research topic into a clinically relevant component of melanoma management. They are now the key to one of the most relevant clinical questions: not simply whether neoadjuvant treatment works, but which patients require different levels of treatment intensity. The current challenge is to identify biomarkers that can predict response, stratify prognosis, and ideally support treatment selection before surgery. This question becomes even more crucial because the available neoadjuvant regimens differ substantially in their toxicity profiles. While combining ipilimumab and nivolumab can induce impressive pathological response rates, it can also be associated with a non-negligible rate of immune-related adverse events. These include induced endocrinopathies, hepatitis, skin toxicity, and colitis. In contrast, single-agent anti-PD-1 approaches may present a safer profile but may represent a less active therapeutic strategy. Therefore, the role of biomarkers could no longer be considered only a prognostic one, but an increasingly practical one. The ultimate objective is to identify, before surgery, those patients who require treatment intensification and those who may achieve durable benefit with less intensive therapeutic strategies. This represents one of the most important future directions in melanoma research, because avoiding unnecessary toxicity without compromising efficacy is becoming just as important as improving patient outcomes [12].

At present, no biomarker is sufficiently validated to guide day-to-day clinical practice. Still, several candidates have shown consistent data throughout, particularly PD-L1, IFN-gamma, tumor mutational burden (TMB), and imaging biomarkers such as FDG-PET/CT. At the same time, one of the major obstacles in the interpretation of the melanoma biomarkers is that a great deal of them present a duality; they tend to behave both as prognostic and as predictive variables. A biomarker that is associated with a favorable immune microenvironment could identify patients with intrinsically better outcomes independently of the therapeutic path chosen, while it could also be used in predicting improved sensitivity to checkpoint blockade. This overlap complicates the interpretation of translational analyses and partially explains why many promising biomarkers have not yet entered routine clinical practice. Another important limitation is represented by the tumor heterogeneity. Different metastatic sites from the same patient may present a variable expression of PD-L1, immune infiltration, and mutational profile. Consequently, single-site biopsies may be poorly representative of the entire disease biology.

Among tissue biomarkers, PD-L1 has been one of the most investigated. Higher PD-L1 expression in pretreatment tumor has been associated in some studies with a greater likelihood of MPR to neoadjuvant checkpoint blockade. However, the signal is not uniform. Responses were recorded in tumors with low or absent PD-L1 expression. In other words, PD-L1 may be informative, but it is not robust enough on its own to decide who should receive a single-agent anti-PD-1 therapy and who should receive ipilimumab and nivolumab. Different studies have made use of different antibodies, scoring systems, and positivity cut-offs, making cross-trial comparisons difficult. Furthermore, the PD-L1 expression is not static over time. It may dynamically change during a treatment or across various treatments and may also be influenced by inflammatory signals within the tumor microenvironment. In addition, as discussed previously, a single baseline biopsy may not fully capture the complexity of the interaction between tumor cells and the immune system. For this reason, PD-L1 alone is increasingly considered insufficient as an isolated and single biomarker, despite remaining one of the most widely explored markers in immunotherapy trials of the past [12].

IFN-γ gene-expression signatures represent another promising biomarker. They reflect the degree of pre-existing inflamed tumor microenvironment, which strongly correlates with antigen presentation, T-cell recruitment, and cytotoxic immune activation. A 2024 biomedicine review reported that elevated baseline IFN-γ-related gene-expression signatures extend beyond static tumor characteristics and may serve as a functional biomarker capturing the readiness of the tumor microenvironment to respond to immunotherapy. Compared with PD-L1, IFN-γ-related signatures may be a better reflection of the global immune status and of the tumor microenvironment rather than the expression of a single protein, namely PD-L1. This is particularly relevant in melanoma, a type of cancer where immune activation is often highly dynamic and spatially heterogeneous. Several translational analyses from neoadjuvant trials have shown that tumors enriched in IFN-γ-associated genes are more likely to demonstrate an increased infiltration by activated CD8+ T cells and a greater probability of achieving MPR when immunotherapy is used. Nevertheless, even the IFN-γ signatures still lack universal standardization and remain mostly research tools [13].

In melanoma, where UV-induced DNA damage contributes to a high mutational load, TMB is biologically plausible as a marker of neo-antigen generation and therefore immunogenicity. In one neoadjuvant dataset, all patients with both high TMB and high IFN-γ achieved a pathological response, whereas the response rate fell to 39% in tumors with both low TMB and low IFN-γ. Intermediate combinations still performed relatively well, with response rates of 89% for high TMB/low IFN-γ and 91% for low TMB/high IFN-γ [8]. TMB should not be interpreted as an absolute surrogate of response. Some tumor types, despite having a high mutational burden still fail to respond to immunotherapy. In fact, durable responses could also be recorded occasionally even in tumors with a lower mutational load. Such observation suggests that the quality of the neoantigens and the immune contexture surrounding each tumor may be just as important as the quantity of mutations itself. Therefore, TMB may achieve its greatest predictive value when it is integrated with inflammatory biomarkers [14,15,16].

The recent 5-year PRADO biomarker analysis reinforced exactly this point. High TMB, high IFN-γ signature, and positive PD-L1 expression were all associated with more favorable outcomes. In patients with high levels of all three biomarkers, an MPR rate approaching 100% and 5-year EFS were recorded. In contrast, those with triple-low expression had only 18% MPR and 41% 5-year EFS. These findings suggest that a composite model integrating PD-L1, TMB, and inflammatory gene expression may be far more clinically useful than any single variable alone [6].

Another emerging area of major interest is circulating tumor DNA (ctDNA) and the minimal residual disease assessment. Unlike the tissue biomarkers discussed before, ctDNA offers the possibility of dynamic and minimally invasive monitoring of disease burden over time. ctDNA consists of fragmented tumor-derived DNA released into the bloodstream, carrying tumor-specific genomic alterations. These alterations can be identified through highly sensitive techniques such as digital droplet PCR and next-generation sequencing. ctDNA may better capture the tumor heterogeneity and clonal evolution when compared with the single tissue biopsy because it can reflect molecular information that comes from multiple tumor sources. Recent evidence suggests that ctDNA may serve as a real-time biomarker capable of detecting molecular relapses, monitoring treatment response, and identifying resistance mechanisms before the conventional radiological progression becomes clinically evident.

One of the most interesting and valuable applications of ctDNA in melanoma is the early detection of minimal residual disease (MRD). Even in cases of apparently complete surgery and radiologic remission, microscopic residual disease may persist. That could later lead to recurrence. In several studies, the postoperative positivity of ctDNA was strongly associated with an increased relapse risk. It also often preceded radiologic recurrence by a margin of several months. In the setting of neoadjuvant treatment, early ctDNA clearance during immunotherapy could be used potentially to identify patients that achieved deep molecular responses even before surgery takes place. Persistent ctDNA positivity may indicate the presence of occult resistant disease despite apparent pathological or radiological response.

ctDNA kinetics may also provide additional information during immunotherapy treatment. A rise in ctDNA levels has been associated with an inferior survival outcome and the presence of early resistance, while rapid decline or a complete clearance generally correlates with improved progression-free and overall survival. ctDNA could also be used as a tool to further distinguish true progression from pseudoprogression, a well-recognized and difficult challenge during checkpoint blockade.

However, the ctDNA is not without limitations. ctDNA sensitivity tends to decrease in low-volume or early-stage disease because of limited tumor shedding. The detection rates may also vary according to the metastatic site. In addition, assay heterogeneity, lack of standardized cut-offs, and variability between detection platforms could still limit widespread clinical implementation in day-to-day clinical practice. Nevertheless, emerging technologies such as fragmentomics, methylation profiling, and tumor-informed sequencing panels are progressively improving the assay sensitivity. Future strategies are focused on integrating ctDNA with pathology results, imaging, and immune-related biomarkers to support a more individualized and adaptive neoadjuvant treatment approach in melanoma [17,18,19,20,21,22,23,24].

Finally, imaging biomarkers also deserve consideration, especially FDG-PET/CT. Imaging is clearly less specific biologically than genomic biomarkers, but it has the advantage of being accessible and repeatable. In the neoadjuvant setting, radiological response by RECIST has shown prognostic value, with 2-year RFS of 100% in complete responders and 96% in pPR in one pooled analysis. But the fact remains that radiological response may underestimate true tumor clearance. PET/CT, therefore, appears most useful not as a substitute for pathology, but as an additional dynamic factor that can complement tissue-based assessment [25,26].

**Figure 3:**
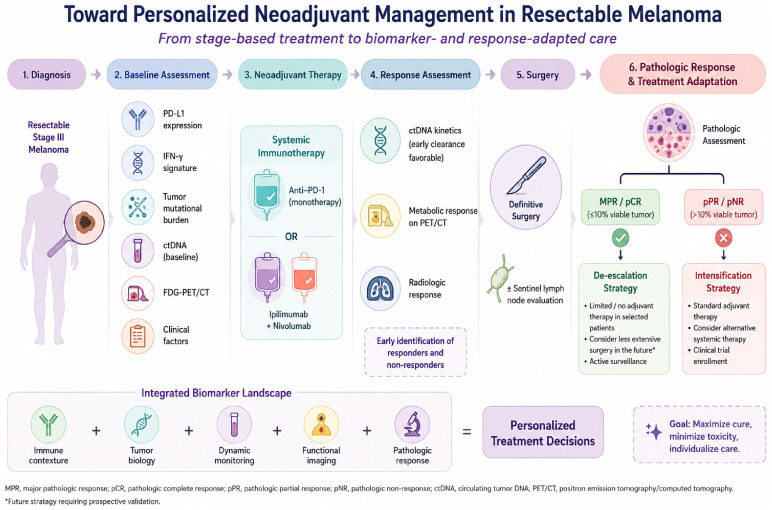
Conceptual Model for Future Treatment Personalization in Resectable Melanoma.

From a clinical perspective, it is important to distinguish between prognostic and predictive biomarkers. Prognostic biomarkers provide information about the expected outcome regardless of treatment. Predictive biomarkers identify patients who are more likely to benefit from a specific therapeutic intervention. In melanoma, this distinction is often challenging because many biomarkers appear to possess both prognostic and predictive properties. For example, an inflamed tumor microenvironment characterized by high PD-L1 expression or an elevated IFN-γ signature may be associated with improved outcomes in general, while also predicting greater sensitivity to immune checkpoint blockade. Overall, current evidence suggests that biomarkers in resectable melanoma are moving from exploratory correlatives to future predictive tools, but they are not yet ready to dictate day-to-day clinical practice. Although the available biomarker data are highly encouraging, a biomarker-driven treatment algorithm cannot yet be recommended in routine clinical practice. At present, none of the proposed biomarkers, namely PD-L1, IFN-γ, TMB, ctDNA, or FDG-PET response, have been prospectively validated as a standalone tool for treatment selection. A potential framework could include baseline tumor biology (PD-L1 expression, IFN-γ signature, TMB), dynamic markers of treatment response (ctDNA kinetics and metabolic response on FDG-PET), and pathological response after surgery. This concept is supported by translational analyses from PRADO, where composite biomarker profiles demonstrated greater predictive value than individual markers alone (Table 2, Figure 3).

## 5. Future Perspectives and Open Questions

Recent randomized studies, particularly SWOG S1801 and NADINA, have provided strong evidence supporting the clinical benefit of neoadjuvant approaches in selected patients with resectable melanoma. The next challenge is to refine patient selection and optimize regimen choice. NADINA clearly favored neoadjuvant ipilimumab plus nivolumab, but it also showed substantially higher grade ≥ 3 toxicity than adjuvant anti-PD-1 alone. By contrast, SWOG S1801 showed benefit with perioperative pembrolizumab and a safer profile. This leaves an important unresolved question: who should receive single-agent anti-PD-1 and who should receive the combination [4]?

This is exactly why current and emerging phase II studies are so relevant. In NEO-MEL-T (NCT04139902), neoadjuvant dostarlimab plus the anti-TIM-3 antibody cobolimab improved MPR compared with dostarlimab alone, suggesting that novel dual-checkpoint combinations beyond CTLA-4 may further improve response rates [27]. In EA6194 (NCT04708418), pembrolizumab plus intratumoral vidutolimod also produced encouraging pRR compared to pembrolizumab alone [28]. Additional encouraging data comes from NeoACTIVATE Arm C. This study demonstrated that neoadjuvant atezolizumab plus tiragolumab, a humanized monoclonal antibody that functions as an immune checkpoint inhibitor by targeting the T-cell immunoreceptor with Ig and ITIM domains (TIGIT) pathway, achieved an MPR in 47.1% of patients, with only 5.9% grade ≥ 3 toxicity [29]. Additional immune checkpoint combinations are also under current investigation. Among them, LAG-3 inhibition has emerged as a potential strategy following the success of relatlimab in advanced melanoma. Recently, a phase II [30] study reported long-term outcomes from evaluating neoadjuvant followed by adjuvant relatlimab plus nivolumab in patients with resectable stage III/IV melanoma. The regimen achieved a MPR of 63%, while updated analyses demonstrated that 80% of patients remained event-free at 4 years and 95% of patients achieving MPR remained recurrence-free. Furthermore, it presented B7-H3 as a potential biomarker of resistance, highlighting additional opportunities for treatment personalization and novel therapeutic combinations.

Still, most of these studies are small phase II trials and often single arm. Despite their practice-changing impact, SWOG S1801 and NADINA leave several clinically relevant questions unanswered, including the optimal management of non-responders and the role of adjuvant treatment after MPR.

An equally important challenge concerns patients who fail to achieve a pathological response. While patients with MPR consistently demonstrate excellent outcomes, those with pNR remain at substantially higher risk of recurrence despite surgery and adjuvant treatment. The optimal management of these patients remains uncertain. Potential strategies include treatment intensification, targeted therapy for BRAF-mutant disease and enrollment in clinical trials. Improving outcomes for non-responders is likely to represent one of the major priorities of future neoadjuvant research.

Ultimately, the future of this field is unlikely to move toward a model in which: “all patients should receive neoadjuvant ipilimumab and nivolumab” but rather toward a more selective model: intensive therapy for those with the highest biological risk, and increasing integration of biomarkers, pathology, and imaging to guide everyday clinical practice.

## 6. Conclusions

The management of resectable stage III melanoma is undergoing a rapid transformation. What was once considered a disease treated primarily through surgery is increasingly being approached as a systemic disease from the time of diagnosis. An important milestone was the development of effective adjuvant therapies with anti-PD-1 agents and BRAF/MEK inhibitors that demonstrated that relapse risk could be significantly reduced after surgery. More recently, neoadjuvant strategies have shown that treatment of the intact tumor can enhance immune activation and potentially improve long-term clinical outcomes.

Trials such as SWOG S1801 and especially NADINA have substantially influenced the current management of resectable melanoma and have provided compelling evidence supporting the clinical value of neoadjuvant immunotherapy in achieving major pathological response rates.

At the same time, several important challenges remain unresolved, including optimal regimen selection, treatment intensity, toxicity management, and patient selection. In this context, biomarkers such as PD-L1, IFN-γ-related signatures, TMB, ctDNA, and pathological response are emerging as promising tools for treatment personalization.

Beyond improving EFS, the most important contribution of the neoadjuvant era may be the shift it has produced in the way resectable melanoma is conceptualized. The focus is no longer limited to reducing recurrence risk but increasingly involves tailoring treatment according to biological response. In this regard, the use of pathological response as a clinically actionable endpoint may represent one of the most important developments in the management of resectable melanoma over the last decade. Ongoing clinical trials continue to explore new combinations and novel immune targets, aiming to improve efficacy while reducing toxicity. Ultimately, the future of melanoma management will likely depend on combining effective systemic therapies with increasingly personalized treatment approaches.

## Figures and Tables

**Table 1:** Key neoadjuvant immunotherapy trials in melanoma: study-specific endpoints and outcomes.

Trial	Neoadjuvant Regimen	Pathologic Response	Survival
NADINA [9]	Ipi (80 mg) + Nivo (240 mg)	59% MPR	84% 12-mo EFS
SWOG S1801 [8]	Pembrolizumab (perioperative)	21% pCR (per review)	72% 2-year EFS
OpACIN-neo [4,5]	Ipi (1 mg/kg) + Nivo (3 mg/kg)	77% pRR	82% 3-year RFS
PRADO [6]	Ipi + Nivo (response-driven)	61% MPR	93% 2-year RFS (for MPR)

Abbreviations: MPR = major pathologic response; pCR = pathologic complete response; pRR = pathologic response rate; EFS = event-free survival; RFS = relapse-free survival. Because the available studies differ in design, population, endpoints, and follow-up duration, the outcomes summarized in this table should be interpreted as study-specific results rather than as directly comparable measures of efficacy. Endpoints differ across studies; therefore, pCR, MPR, pRR, EFS, and RFS should not be interpreted as interchangeable or directly comparable outcomes.

**Table 2:** Promising biomarkers guiding neoadjuvant therapy decision-making in resectable melanoma.

Biomarker	Type	Biological Rationale	Evidence in Neoadjuvant Trials	Clinical Relevance	Limitations
PD-L1 expression	IHC	Pre-existing immune activation	Associated with higher pathologic response in several trials	May guide therapy intensity	Limited specificity
IFN-γ signature	Gene expression	Reflects inflamed tumor microenvironment	Correlates with major pathologic response	Promising predictive biomarker	No standardized cut-offs
Tumor mutational burden	Genomic	Higher neoantigen load	Improved response when combined with IFN-γ	Useful in composite models	Weak alone
Pathologic response (MPR/pCR)	Histopathology	Direct measure of tumor eradication	Strong correlation with RFS	Best surrogate endpoint	Post-surgical only
FDG-PET response	Imaging	Measures metabolic activity	Correlates with survival outcomes	Useful preoperative assessment	Imperfect concordance with pathology
Composite biomarkers	Integrated	Combines immune activation + mutational load	High predictive value in translational analyses	Future direction	Needs validation

## Data Availability

No new data was created.
